# Application of the Western-based adjuvant online model to Korean colon cancer patients; a single institution experience

**DOI:** 10.1186/1471-2407-12-471

**Published:** 2012-10-12

**Authors:** Minkyu Jung, Geon Woo Kim, Inkyung Jung, Joong Bae Ahn, Jae Kyung Roh, Sun Young Rha, Hyun Cheol Chung, Nam Kyu Kim, Tae Il Kim, Sang Joon Shin

**Affiliations:** 1 Division of Medical Oncology, Department of Internal Medicine, Yonsei University College of Medicine, 50 Yonsei-ro, Seodaemun-go, Seoul, 120-752, Korea; 2 Department of Mathematics, Yonsei University College of Science, Seoul, Korea; 3 Department of Biostatistics, Yonsei University College of Medicine, 50 Yonsei-ro, Seodaemun-go, Seoul, 120-749, Korea; 4 Department of Surgery, Yonsei University College of Medicine, 50 Yonsei-ro, Seodaemun-go, Seoul, 120-752, Korea; 5 Department of Gastroenterology, Yonsei University College of Medicine, 50 Yonsei-ro, Seodaemun-go, Seoul, 120-752, Korea

**Keywords:** Therapy, Adjuvant, Colonic neoplasms, Prognosis

## Abstract

**Background:**

Adjuvant Online (AOL) is web-accessible risk-assessment model that predicts the mortality and the benefits of adjuvant therapy (http://www.newadjuvantonline.com). AOL has never been validated for Asian colon cancer patients.

**Methods:**

Using the Yonsei Tumor Registry database, patients who were treated within the Yonsei University Health System between 1990 and 2005 for T1-4, N0-2, and M0 colon cancer were included in the calculations for survival. Observed and predicted 5-year overall survival was compared for each patient.

**Results:**

The median age of the study population of 1431 patients was 60 years (range, 15–87 years), and the median follow-up duration was 7.9 years (range, 0.06–19.8 years). The predicted 5-year overall survival rate (77.7%) and observed survival (79.5%) was not statistically different (95% Confidential interval, 76.3–81.5) in all patients. Predicted outcomes were within 95% confidential interval of observed survival in both stage II and III disease, including most demographic and pathologic subgroups. Moreover, AOL more accurately predicted OS for patients with stage II than stage III.

**Conclusions:**

AOL tended to offer reliable prediction for 5-year overall survival and could be used as a decision making tool for adjuvant treatment in Korean colon cancer patients whose prognosis is similar to other Asian patients.

## Background

Colon cancer is the third most common cancer in Western countries, and its incidence is rapidly increasing in Asia [1]. The main prognostic factor for survival after surgery for localized disease is tumor stage [2]. Approximately 40% of colon cancer patients have lymph node involvement and 20% have node negative, T3, or T4 disease [3]. Adjuvant chemotherapy after surgery for stage III colon cancer has become a standard therapy and is credited for an estimated 33% reduction in the risk of colon cancer recurrence [4,5]. However, the prognosis of survival for stage II colon cancer is different from that of stage III, and the benefit of adjuvant therapy for stage II remains unclear [2]. Therefore, recommendations for adjuvant treatment in colon cancer patients are based on the clinician’s estimated risk assessment for colon cancer relapse or death and the likely benefit of the therapy.

However, predicting the absolute benefit of adjuvant systemic therapy for an individual patient with colon cancer is complex. To solve this problem, two decision support tools have been developed [6,7]. Adjuvant! Online (AOL, http://www.newadjuvantonline.com) is a computerized, web-accessible, risk assessment model that predicts mortality, recurrence risk, and benefit of adjuvant therapy for Western patients with colon cancer. The program provides estimates for 5-year overall survival (OS), colon cancer-specific survival (CCSS), and disease-free survival (DFS) based on the patient’s age, sex, comorbidities, T stage, tumor grade, nodal status, and adjuvant chemotherapy. AOL was developed based on information from the Surveillance, Epidemiology and End Results (SEER) registry [6].

Recently, this model has been externally validated in Western patients with colon cancer and acceptable prediction for survival of patients with stage III was observed [8]. However, this model has never been validated in Asian colon cancer patients, whose characteristics are different from Western patients. Some studies have reported ethnic differences in tumor stage and survival. Among non-Hispanic Whites, non-Hispanic Blacks, Hispanics, and Asian/Pacific Islanders, Blacks were more likely than Whites to have advanced stage disease and Asians/Pacific Islanders had a lower risk of death from colorectal cancer in the same stage [9-12].

Therefore, the aim of this study was to evaluate whether Western based prognostic model, AOL, could be a useful tool in Korean colon cancer patients whose prognosis is similar to other Asian patients.

## Methods

### Patients

Patients who were treated at the Yonsei University Health System (YUHS) between 1990 and 2005 and were identified in the Yonsei Tumor Registry (YTR) database were included in the study if they met the following criteria: complete resection of colon cancer, with no evidence of distant metastasis on initial work-up or on surgical exploration, and all lesions located between the cecum and the rectosigmoid. Based on AOL criteria, clinicopathological variables collected in this study included age, sex, T stage, number of examined lymph nodes, number of positive lymph nodes, histologic grade, and treatment with adjuvant chemotherapy. Patients with previous malignant disease; those who received neoadjuvant therapy; or those who they had unknown tumor size, nodal status, or adjuvant systemic therapy status were excluded. The study was approved by the YUHS institutional review board (IRB number: 4-2010-0178).

### Treatment protocol

Adjuvant chemotherapy was administered to patients with adjuvant chemotherapy was administered to patients with node positive or T3-4 tumors. Adjuvant chemotherapy was a 5-fluourouracil (5-FU) based regimen that was administered for 6 months.

### Data analysis

The aim of this study was comparisons between the predicted and observed OS. The observed OS was measured from the date of diagnosis to the date of death. The observed outcome for each patient was obtained from the YTR database and Korea National Statistical Office (KNSO). Using the same patient population, the predicted 5-year OS values were derived for each patient using the standard AOL version available in October 2010. The input options for AOL were age, sex, comorbidity, depth of invasion, positive nodes, examined nodes, histologic grade, and adjuvant systemic therapy. The default comorbidity assumption of “minor health problems” was used, since we could not retrieve reliable comorbidity data from YTR.

### Statistical analysis

The observed 5-year OS was compared using the method of Kaplan-Meier (KM) with predicted estimate from AOL. For the same datasets, the average predicted OS was calculated from individual predicted outcomes by AOL. The observed and predicted survivals were compared by descriptive manner using the absolute difference. If predicted values were within 95% confidential interval of observed OS, we considered AOL to accurately predict OS. The AOL predictions were divided into 5% intervals, and intervals were grouped so that each interval contained at least 50 patients. The observed KM estimations for each interval subset were plotted against the average prediction for AOL. All analyses were performed using SAS version 9.2 (SAS Inc., Cary, NC) and R statistical software.

## Results

### Patient characteristics

Among 1598 patients with T1-4, N0-2, M0 colon cancer diagnosed at YUHS, 1431 (89.5%) met our eligibility criteria. For this study, we excluded synchronous colon cancer (n = 20), unknown T stage or nodal status (n = 21), or less than 5 years of follow-up duration (n = 126). The median age was 60 years (range, 15–87 years). Among all study patients, 81.6% had T3 stage and 64% had no lymph node metastasis. More than 10 lymph nodes were harvested in 84% patients. Of all, 765 patients (53.4%) underwent surgery and received adjuvant 5-FU chemotherapy. The 420 (45.6%) of 921 patients treated with chemotherapy were node negative and T3 or T4 tumor. And 165 (32.5%) of 510 patients who had lymph node-positive did not receive chemotherapy. Among 165 patients, 43 patients did not receive chemotherapy due to old age, 37 patients due to poor performance status, 61 patients due to postoperative complication and 24 patients refused chemotherapy. Over the median follow-up duration of 7.9 years (range, 0.06–19.8 years), 448 of 1431 patients (31.3%) died before October 31, 2010. Among them, 292 patients (65.2%) died due to colon cancer and 123 (27.5%) died unrelated to cancer. There were 33 cases (7.4%) of metachronous second primary cancer related deaths.

### Application of AOL to Korean patients with surgery alone

**Figure 1:**
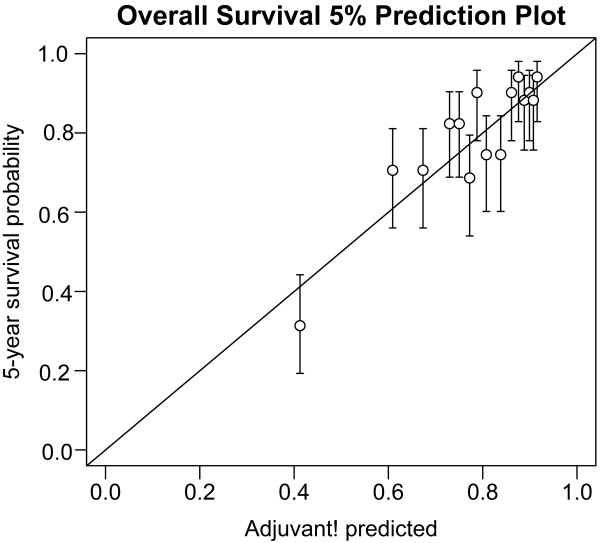
Comparison of AOL predicted and observed 5-year survival in patients with underwent surgery alone.

For all patients (n = 1431), the predicted 5-year overall survival rate (77.7%) and observed survival (79.3%) was not statistically different (95% Confidential interval [CI], 77.3–81.5). Table 1 shows comparisons between AOL predictions and observations for 5-year KM rate with 95% CI based on demographic and pathologic parameters in patients who underwent surgery alone (n = 666). The predicted survival differed from the median observed 5-year survival rate by 2.7%; however, predicted survival rate was within 95% CI of the KM estimate. For lymph node- negative, stage II subgroup (75.2%), AOL exactly estimated OS with difference rate by 0.9%. In addition, AOL estimates were within the observed 95% CI for OS in all subgroups (20 of 21 subgroups), except two subgroups. AOL underestimated OS in patients younger than age of 50 (predicted-observed = −8.3%), and these predictions were outside the 95% CI of KM estimate, and patients with more than 10 lymph nodes examined. Figure 1 shows the relationship between predicted and observed OS divided into 5% intervals in patients with surgery alone.

**Table 1:** Baseline characteristics and 5-year survival predicted and observed in the patients with surgery alone

			**Mean % of 5-year survival**		**% Delta (Pred-Obs)**
	**Number**	**%**	**AOL predicted**	**YTR observed (95% CI)**	
All patients	666	100	77.07	79.73 (76.47, 82.59)	−2.66
Age					
< 50	107	16.1	83.31^*^	91.59 (84.46, 95.53)	−8.28
50-59	148	22.2	80.61	83.78 (76.79, 88.82)	−3.17
60-69	227	34.1	78.34	80.17 (74.37, 84.8)	−1.83
≥ 70	184	27.6	69.03	69.01 (61.78, 75.15)	0.02
Sex					
Male	400	60.1	76.19	78.75 (74.41, 82.44)	−2.56
Female	266	39.4	78.40	81.2 (75.97, 85.41)	−2.8
T stage					
T1	79	11.9	91.08	94.94 (87.07, 98.07)	−3.86
T2	117	17.5	85.76	83.76 (75.73, 89.32)	2
T3	456	68.5	72.83	76.97 (72.82, 80.57)	−4.14
T4	14	2.1	63.57	50 (22.86, 72.21)	13.57
Number of positive nodes					
0	501	75.2	83.92	84.83 (81.38, 87.69)	−0.91
1-3	111	16.7	62.65	67.56 (57.99, 75.4)	−4.91
4-10	47	7.1	46.79	59.57 (44.21, 71.99)	−12.78
> 10	7	1.0	19.00	42.86 (9.78, 73.44)	−23.86
Number of examined nodes					
1-3	27	4.1	85.56	88.89 (69.39, 96.27)	−3.33
4-10	122	18.3	78.75	72.13 (63.26, 79.21)	6.62
> 10	517	77.6	76.23^*^	81.04 (77.39, 84.17)	−4.81
Histologic grade					
1	110	16.5	81.67	82.73 (74.27, 88.62)	−1.06
2	455	68.3	75.77	78.9 (74.85, 82.37)	−3.13
3	32	4.8	72.59	81.25 (62.95, 91.11)	−8.66
Undefined	69	10.4	80.38	79.71 (68.16, 87.45)	0.67

^*^This predicted estimate is outside the 95% CI of the Kaplan-Meier estimate.

*AOL*: Adjuvant Online; *YTR*: Yonsei Tumor registry; *CI*: confidential interval; *Pred*: predicted; *Obs*: observed.

### Application of AOL to Korean patients underwent surgery plus 5-FU chemotherapy

**Figure 2:**
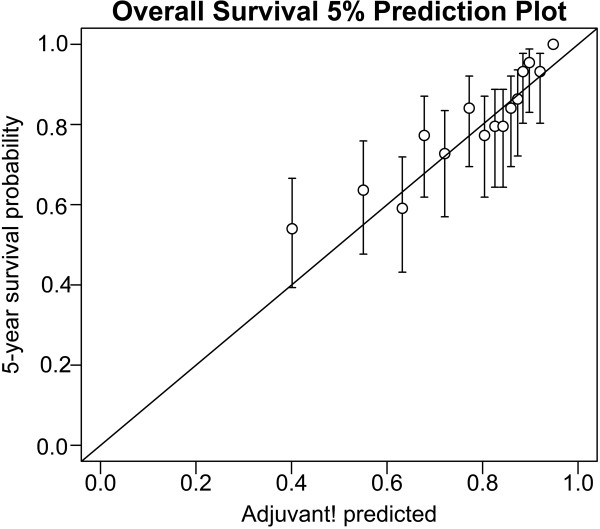
Comparison of AOL predicted and observed 5-year survival in patients treated with surgery and chemotherapy.

In Table 2 presents the overall and subgroup univariate mean predictions for AOL and the observed OS in patients who underwent surgery and received 5-FU based chemotherapy. The difference between estimates and observed 5-year OS rate in all patients (n = 765) was 1.2%. Except three subgroups which had small number of patients, predicted OS in all subgroup were in 95% CI of the observed OS by KM estimate. For patients with stage II (n = 420), AOL demonstrated excellent prediction accuracy for OS, differing by 0.4%. Figure 2 illustrated AOL average predicted estimates for 5-year OS compared with the observed KM estimates.

**Table 2:** Baseline characteristics and 5-year survival predicted and observed in the patients with surgery plus chemotherapy

			**Mean % of 5-Year outcomes**		**% Delta (Pred-Obs)**
	**Number**	**%**	**AOL predicted**	**YTR observed (95% CI)**	
All patients	765	100	78.19	79.35 (76.3, 82.05)	−1.16
Age					
< 50	177	23.1	83.37	87.57 (81.74, 91.63)	−4.2
50-59	226	29.5	79.56	80.09 (74.26, 84.73)	−0.53
60-69	270	35.3	76.51	76.3 (70.76, 80.93)	0.21
≥ 70	92	12.1	69.75	70.65 (60.2, 78.84)	−0.9
Sex					
Male	450	58.8	77.57	78.67 (74.59, 82.17)	−1.1
Female	315	41.2	79.06	80.32 (75.48, 84.3)	−1.26
T stage					
T1	4	0.5	89.50	75 (12.79, 96.05)	14.5
T2	20	2.6	82.35	85 (60.38, 94.9)	−2.65
T3	712	93.1	78.78	80.48 (77.37, 83.2)	−1.7
T4	29	3.8	59.21	48.28 (29.47, 64.78)	10.93
Number of positive node					
0	420	54.9	86.87	86.43 (82.77, 89.36)	0.44
1-3	242	31.6	74.31	77.27 (71.46, 82.05)	−2.96
4-10	79	10.3	57.24^*^	69.62 (58.19, 78.49)	−12.38
> 10	24	3.2	34.25^*^	8.33 (1.44, 23.3)	25.92
Number of examined nodes					
1-3	3	0.4	79.33^*^	100 (100, 100)	−20.67
4-10	83	10.9	76.77	77.11 (66.49, 84.74)	−0.34
> 10	679	88.7	78.35	79.53 (76.29, 82.37)	−1.18
Histologic grade					
1	94	12.3	82.44	81.91 (72.53, 88.35)	0.53
2	561	73.3	78.52	79.14 (75.54, 82.28)	−0.62
3	44	5.8	69.50	72.73 (57, 83.49)	−3.23
Undefined	66	8.6	75.09	81.82 (70.21, 89.24)	−6.73

^*^This predicted estimate is outside the 95% CI of the Kaplan-Meier estimate.

*AOL*: Adjuvant Online; *YTR*: Yonsei Tumor registry; *CI*: confidential interval; *Pred*: predicted; *Obs*: observed.

## Discussion and conclusions

In Western population and clinical trial based validation, AOL had reliable prediction of OS for colon cancer patients [8]. This study was performed to test whether AOL prediction of survival is applicable for the Korean colon cancer patients, whose prognosis is similar to other Asian patients [9,13,14].

In the current study, AOL had acceptable prediction for OS for all patients and almost every subgroup. Especially, AOL more accurately predicted OS for patients with stage II than stage III. Gil et al. [8] reported that AOL had acceptable reliability for patients with stage III disease and tended to overestimate survival for patients with stage II disease who received 5-FU from the population-based data.

What are the reasons for inverse validation between Western and Korean population? One possible explanation is ethnic difference. Asian colon cancer patients tend to experience better survival than Western patients in the same stage [9,11,12,15]. Our data showed that the observed OS was better than that predicted by AOL, although there was no statistical difference. Especially, AOL overestimated survival by 8.3% in patients younger than 50 years. Therefore, AOL tended to overestimate survival for Western patients with stage II disease treated with 5-FU and exactly predicted survival for Korean cancer patients with stage II.

The other possible reason of different validation is different subgroup between two populations. The median age of Korean population was 8-year younger than the Western population. Interestingly, the median age of the clinical trial cohort of Western patients was 64 years old, which is younger than population cohort of Western patients. In both Gil et al. and current study, the default comorbidity assumption of “minor health problems” was used, since we could not retrieve reliable comorbidity data from each cohort. Accordingly, Western population cohort included more old age patients and high risk comorbidity patients than the trial based cohort. AOL overestimated survival in the western population cohort and similarly estimated in the clinical trial cohort. In contrast to this, Korean population cohort included more young age and low risk comorbidity patients than the Western population, which AOL accurately predicted survival for patients in stage II and III.

The last possible reason for the difference in predicted and observed outcomes can also be explained by the limitation of the AOL estimation regarding risk reduction by chemotherapy. Estimates of prognosis are mainly based on the SEER estimates of outcome for colon cancer patients in the general population. The efficacy of therapy is estimated based on the proportional risk reductions that which were obtained from meta-analyses of the effectiveness of adjuvant therapy and from the data published or presented from individual randomized clinical trials [6]. Therefore, AOL tended to overestimate survival of Western colon cancer patients, and more accurately predicted in Korean patients whose survival are better than the Western patients.

Many studies have reported the number of evaluated lymph nodes (ELN) was positively associated with survival of colon cancer patients with not only stage II, but stage III [16]. However, some studies reported that the number of ELN was positively correlated with survival in stage II, but did not affect the long-term outcome in stage III [13,17]. In the current study, no significant association observed between ELN and survival. Kaplan-Meier analyses demonstrated 5-year overall survival rates for the number of ELN 1–3, 4–10, and > 10 of 90%, 73%, and 80%, respectively (Additional file 1: Table S1). A possible reason why the patients with less than 4 of ELN had the most favorable survival might be that these patients had favorable variables than the other patients, such as higher portion of younger age, low T stage, less number of positive node, and lower histologic grade (Additional file 2: Figure S1). In addition, the number of patients with less than 4 of ELN was only 30 (2.1%). Except for these patients, the number of ELN had positive trend of good survival in our study.

This study had several limitations. First, tumor grade was not available for some of our cases (9.4%), and the information of comorbidity could not be checked because the YTR is a retrospective database. Second, our study examined a time period when the standard chemotherapy was a 5-FU-based chemotherapy, the currently used oxaliplatin-based chemotherapy in patients with stage III and high risk stage II, although predicted survival used by AOL recommendation for the benefit of 5-FU based chemotherapy. Third, we had insufficient clinicopathological parameters such as preoperative carcinoembryonic antigen level [18], lymphovascular involvement [19], microsatellite instability [20], and other molecular markers [21].

In conclusion, we found that the AOL prediction system, which is based on Western patients, is suitable for Korean colon cancer patients with not only stage II but also stage III. Therefore, AOL which is a easily accessed tool, provides important information for the physician in terms of survival for Korean and Asian colon cancer patients, whose disease patterns and survival are similar [9,13,14].

## Abbreviations

AOL: Adjuvant online; OS: Overall survival; CCSS: Colon cancer-specific survival; DFS: Disease-free survival; SEER: Surveillance, epidemiology and end results; YUHS: Yonsei University Health System; YTR: Yonsei Tumor Registry; 5-FU: 5-Fluourouracil; KNSO: Korea National Statistical Office; KM: Kaplan-Meier; CI: Confidential interval; Pred: Predicted; Obs: Observed.

## Competing interests

The authors declare that they have no competing interests.

## Authors’ contributions

MJ and SJS was responsible for drafting the manuscript and GWK and IJ contributed to analysis and interpretation of data. JBA, JKR, NKK, WHK, and SJS contributed to acquisition of data. SYR and HCC participated in its design and coordination and helped to draft the manuscript. All authors read and approved the final manuscript.

## Pre-publication history

The pre-publication history for this paper can be accessed here:

http://www.biomedcentral.com/1471-2407/12/471/prepub

## Supplementary Material

Additional file 1 **Table S1.** Baseline characteristics according to number of examined nodes (n=1431). Click here for file

Additional file 2 **Figure S1.** Observed overall survival according to number of examined nodes by Kaplan-Meier curve. Click here for file
