# Supplementary material for: Application of the Western-based adjuvant online model to Korean colon cancer patients; a single institution experience
Source: BMC Cancer. 2012 Oct 12;12:471. doi: 10.1186/1471-2407-12-471 (PMC3534402; doi:10.1186/1471-2407-12-471)
Supplement: Additional file 1 — Table S1. Baseline characteristics according to number of examined nodes (n=1431). [file 1471-2407-12-471-S1.docx]

**Figure legend**

**Additional figure 1 Observed overall survival according to number of examined nodes by Kaplan-Meier curve.**

**Additional table 2. Baseline characteristics according to number of examined nodes (n=1431)**

|  |  | Number of examined nodes (%) | | | p-value |
| --- | --- | --- | --- | --- | --- |
|  |  | 1-3 | 4-10 | >10 |  |
| Age |  |  |  |  | 0.001 |
|  | Median (Interquartile range) | 57 (40-74) | 63 (48-78) | 60 (44-76) |  |
| Sex |  |  |  |  | 0.189 |
|  | Male | 13 (43.3) | 124 (60.5) | 713 (59.6) |  |
|  | Female | 17 (56.7) | 81 (39.5) | 483 (40.4) |  |
| T stage |  |  |  |  | <0.001 |
|  | T1 | 15 (50) | 34 (16.6) | 34 (2.8) |  |
|  | T2 | 4 (13.3) | 29 (14.1) | 104 (8.7) |  |
|  | T3 | 11 (36.7) | 137 (66.8) | 1020 (85.3) |  |
|  | T4 | 0 | 5 (2.4) | 38 (3.2) |  |
| Number of positive node | |  |  |  | <0.001 |
|  | 0 | 28 (93.3) | 154 (75.1) | 739 (61.8) |  |
|  | 1-3 | 2 (6.7) | 42 (20.5) | 309 (25.8) |  |
|  | 4-10 | 0 | 9 (4.4) | 117 (9.8) |  |
|  | > 10 | 0 | 0 | 31 (2.6) |  |
| Histologic grade | |  |  |  | 0.004 |
|  | 1 | 11 (36.7) | 38 (18.5) | 155 (13) |  |
|  | 2 | 14 (46.7) | 142 (69.8) | 860 (84.6) |  |
|  | 3 | 1 (3.3) | 8 (3.9) | 67 (5.6) |  |
|  | Undefined | 4 (13.3) | 17 (1.2) | 114 (9.5) |  |
