# Supplementary figures and images for: Application of the Western-based adjuvant online model to Korean colon cancer patients; a single institution experience
Source: BMC Cancer. 2012 Oct 12;12:471. doi: 10.1186/1471-2407-12-471 (PMC3534402; doi:10.1186/1471-2407-12-471)

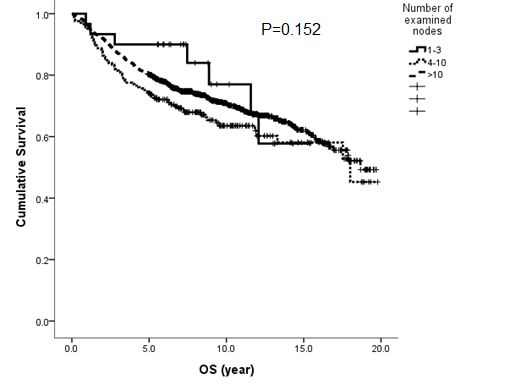

Supplement: Additional file 2 — Figure S1. Observed overall survival according to number of examined nodes by Kaplan-Meier curve. [file 1471-2407-12-471-S2.tiff]
